# GC-elements controlling *HRAS* transcription form *i*-motif structures unfolded by heterogeneous ribonucleoprotein particle A1

**DOI:** 10.1038/srep18097

**Published:** 2015-12-17

**Authors:** Giulia Miglietta, Susanna Cogoi, Erik B. Pedersen, Luigi E. Xodo

**Affiliations:** 1 Department of Medical and Biological Sciences, P.le Kolbe 4, 33100 Udine, Italy; 2 Nucleic Acid Center, Institute of Physics and Chemistry, University of Southern Denmark, DK-5230 Odense M, Denmark

## Abstract

*HRAS* is regulated by two neighbouring quadruplex-forming GC-elements (*hras*-1 and *hras*-2), located upstream of the major transcription start sites (doi: 10.1093/nar/gku 5784). In this study we demonstrate that the C-rich strands of *hras*-1 and *hras*-2 fold into *i*-motif conformations (*i*Ms) characterized under crowding conditions (PEG-300, 40% w/v) by semi-transitions at pH 6.3 and 6.7, respectively. Nondenaturing PAGE shows that the *HRAS* C-rich sequences migrate at both pH 5 and 7 as folded intramolecular structures. Chromatin immunoprecipitation shows that hnRNP A1 is associated under *in vivo* conditions to the GC-elements, while EMSA proves that hnRNP A1 binds tightly to the *i*Ms. FRET and CD show that hnRNP A1 unfolds the *i*M structures upon binding. Furthermore, when hnRNP A1 is knocked out in T24 bladder cancer cells by a specific shRNA, the *HRAS* transcript level drops to 44 ± 5% of the control, suggesting that hnRNP A1 is necessary for gene activation. The sequestration by decoy oligonucleotides of the proteins (hnRNP A1 and others) binding to the *HRAS i*Ms causes a significant inhibition of *HRAS* transcription. All these outcomes suggest that *HRAS* is regulated by a G-quadruplex/*i*-motif switch interacting with proteins that recognize non B-DNA conformations.

The *HRAS* oncogene encodes for a 21-k*D* GTP-ase conveying signals to the nucleus that stimulate cell proliferation^1^. In many tumours *HRAS* is mutated, normally in exon 1, codon 12, 13 or 61, and encodes for an altered protein which constitutively activates downstream pathways causing normal cells to become cancerous cells^2^. In previous works, we have demonstrated that *HRAS* is regulated by two neighbouring GC-rich elements that we called *hras*-1 (nt 432-464, A.N. J00277) and *hras*-2 (nt 509-530, A.N. J00277), located immediately upstream of the major transcription start sites (TSS’s), each capable of folding into a G-quadruplex structure^3^,^4^. By site-directed mutagenesis of the GC-elements, we found that the G-quadruplexes behave as transcription repressors^3^. Under normal conditions, *hras*-1 and *hras*-2 are folded into G-quadruplexes, thus locking the promoter into an inactive state characterized by a low transcription level^3^. Transcription is activated when the G-quadruplexes are unfolded, and the G-elements transformed into canonical B-DNA forms. We found that MAZ, a zinc-finger transcription factor recognizing blocks of guanines, interacts with the promoter GC-elements under cellular conditions^3^. MAZ is an essential protein for gene expression, as it unfolds the *HRAS* G-quadruplexes and activates transcription^3^,^4^. Our data support a transcription model according to which the two neighbouring G-quadruplexes behave as a molecular switch that controls gene expression.

In the present work we interrogated if the complementary C-rich strands of *hras*-1 and *hras*-2 (namely *hras*-1^Y^ and *hras*-2^Y^) fold into the well known *i*M conformation^5^,^6^,^7^,^8^,^9^,^10^,^11^,^12^,^13^,^14^,^15^. We found that *hras*-1^Y^ and *hras*-2^Y^ assume the *i*M conformation under slightly acidic conditions, which are close to neutrality in the presence of a crowding agent, for example PEG-300^16^. We also discovered that the *HRAS i*Ms are recognized by nuclear proteins, including nuclear factor hnRNP A1. This protein, which shows a binding preference for cytosines, unfolds the *i*M conformation of the *HRAS* sequences. When hnRNP A1 was knocked out in T24 bladder cancer cells by a specific shRNA, the level of *HRAS* transcript also dropped to 44 ± 5% of the control. Together, our data provide evidence that hnRNP A1, with its unfolding activity against the *i*M, is an essential factor for the activation of *HRAS*. Indeed, when hnRNP A1 was sequestered by decoy oligonucleotides mimicking the *i*M, *HRAS* transcription was significantly downregulated. The outcome of this work support the notion that *HRAS* expression is regulated by a G-quadruplex/*i*M switch that is controlled by proteins.

## Results and Discussion

**Figure 1:**
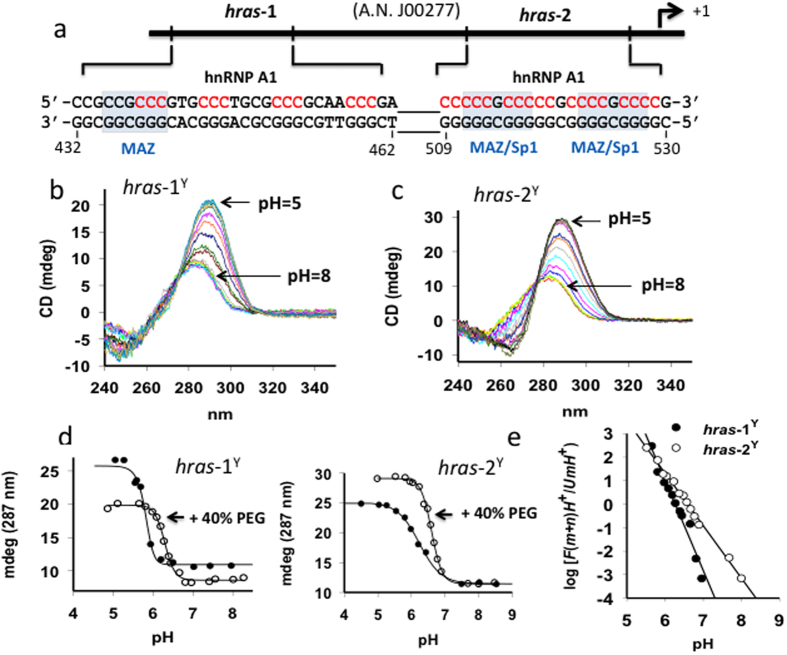
(**A**) Sequences of the GC-rich elements located in the *HRAS* promoter upstream of major TSS’s; (**B,C**) Circular dichroism titrations of *hras*-1^Y^ and *hras*-2^Y^ (3 μM, 1 cm pathlength cell) in 50 mM Tris-acetate, 50 mM KCl, 40% PEG-300 and pH from 4.5 to 8; (**D**) Ellipticity (287 nm) *versus* pH curves for *hras*-1^Y^ and *hras*-2^Y^ in the presence and absence of PEG-300; (**E**) Determination of number of protons picked up by *hras*-1^Y^ and *hras*-2^Y^ upon folding into the *i*M.

The sequence of the *HRAS* promoter immediately upstream of the major transcription start sites is reported in Fig. 1A. It contains two GC-rich elements, *hras*-1 and *hras*-2, composed of blocks of guanines and capable of folding into G-quadruplex structures. In previous works we have demonstrated that these sequences behave as a regulatory switch controlling gene expression^3^,^4^. Such a mechanism has been proposed for other relevant oncogenes including *KRAS*^17^,^18^*, CKIT*^19^,^20^, and *CMYC*^21^,^22^. A couple of comprehensive reviews on this subject have been reported^23^,^24^. In this work we have focused on the complementary C-rich strands *hras*-1^Y^ and *hras*-2^Y^ and have investigated if they fold into stable *i*Ms.

### *i*M formation by the *HRAS* C-rich sequences

To find out if *hras*-1^Y^ and *hras*-2^Y^ can assume the *i*M conformation, we performed circular dichroism (CD) experiments as a function of pH, in 50 mM KCl, 50 mM Tris-acetate, 25 °C. To mimic the crowding conditions of the cell, we analysed the sequences both in the presence and absence of 40% (w/v) PEG-300^16^. Typical CD titrations are shown in Fig. 1B,C. It can be seen that the spectra of *hras*-1^Y^ and *hras*-2^Y^ change dramatically as the pH is gradually decreased from 8 to 4.5. Under acidic conditions (pH 5) both sequences exhibit the characteristic enhanced ellipticity at ~287 nm of a classical *i*M^10^,^12^,^25^,^26^,^27^, while at pH 8 the sequences exhibit a much lower ellipticity, shifted at ~285 nm. By plotting the 287-nm ellipticity as a function of pH, we obtained for each sequence, in the presence or absence of PEG-300, sigmoidal curves reflecting *i*M formation (Fig. 1D). The crowding agent drives the folding at higher pH values: the semi-transition of *hras*-1^Y^ increases from pH 5.9 to 6.3, whereas that of *hras*-2^Y^ increases from pH 6.2 to 6.7. These plots suggest that the *i*Ms are stable in a slightly acidic medium. However, under cellular conditions the *i*M can be stabilized by: (i) transcriptionally induced DNA superhelicity^28^,^29^; (ii) more effective cellular crowding conditions^30^,^31^; (iii) an increased intracellular acidity generated by an increase of the glucose-lactate flux^32^,^33^.

As the ellipticity-*versus*-pH curves are reversible, we evaluated the number of protons involved in the folding of *hras*-1^Y^ and *hras*-2^Y^, by considering the following equilibria:


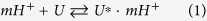



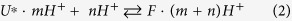


where equilibrium 1 takes into consideration the fact that the unfolded C-rich sequence, *U*, may bind *m* protons before folding into the *i*-motif (as the pKa of cytosine is ∼4.4^34^, at pH 5 about 3 cytosines out of 12 are expected to be protonated); U*·mH^+^ is the unfolded sequence with *m* bound protons. The bases in U*·mH^+^ are assumed to be unstacked, so the formation of this species is accompanied by a negligible change in the CD spectrum^35^. In contrast, upon folding into *i-*motif F, U*·mH^+^ assumes in a cooperative manner other *n* protons to form (*n*+*m*) CH^+^:C base pairs that are stacked in the structure. A significant CD spectral change is expected for the formation of the *i*M^8^,^10^ (equilibrium 2), whose equilibrium constant is *K*_D_ = ([*F·*(m+n)*H*^+^]/([U*·m*H*^+^][*H*^+^]^n^) (3). From the CD plots of Fig. 1B,C, we determined for each sequence the fraction of folded and unfolded species and ratio [*F(n+m)H*^+^]/[ U*·mH^+^]. By plotting log [*F(n+m)H*^+^]/[ U*·mH^+^] *versus* pH, we obtained a straight line whose slope is *n* (Fig. 1E). We obtained values of *n* ∼ 4 and *n* ∼ 2 for *hras*-1^Y^ and *hras*-2^Y^, respectively. This suggests that when *hras*-1^Y^ and *hras*-2^Y^ fold into the *i*M, they assume 4 and 2 protons, respectively, which agrees with the fact that the sequences are partially protonated before folding. A similar behaviour has been previously observed for the formation of the *i*-motif by (C_3_TA_2_)_4_^14^.

### PAGE and analyses of melting curves

**Figure 2:**
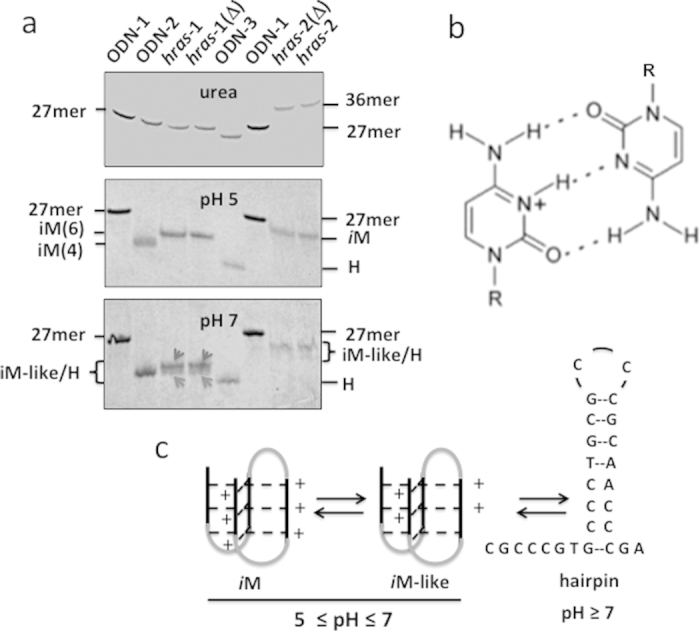
(**A**) PAGE of *hras*-1^Y^ and *hras*-2^Y^ and variants ODN-1, ODN-2 and ODN-3 in denaturing conditions (7 M urea); native conditions at pH 5 and 7. The oligonucleotide sequences are reported in Methods. The electrophoresis was run at 20 °C, the bands stained with stains-all. Δ = heated 10 min 90 °C; (**B**) CH^+^:C base pair; (**C**) Putative equilibrium between *i*Ms involving different protonation levels and a flexible hairpin formed by *hras*-1^Y^ at pH ≥ 7.

The intramolecular *i*M, being a folded structure, migrates in a polyacrylamide gel faster than an unfolded oligonucleotide of the same length. We analysed *hras*-1^Y^ and *hras*-2^Y^ by PAGE under different pH conditions. The mobility of the two C-rich sequences was compared with that of *hras*-1^Y^ variants: ODN-1 (unable to form any structure); ODN-2 (forming an *i*M with 4 CH^+^:C) and ODN-3 (forming a stable W.C. hairpin) (Fig. 2A,B) (Methods). Under denaturing conditions (7 M urea), the 27-mer oligonucleotides (*hras*-1^Y^ and variants) exhibited the same mobility, with the exception of ODN-3 that forms a hairpin even in the presence of 7 M urea. Sequence *hras*-2^Y^, being embedded in a 36-mer oligonucleotide, migrates slowly (Methods). In contrast, under native conditions at pH 5, *hras*-1^Y^, that assumes the *i*M according to CD, migrates with a sharp band faster than that of ODN-1. Interestingly, when the *hras*-1^Y^ sequence is modified to fold into a hairpin stabilized by a stem of 8 W.C. bps (ODN-3) (S_1_), it migrates even quicker than the *i*M. This is because the 6 positive charges of *i*M reduce its negative charge density. The folding of *hras*-1^Y^ into the *i*M is quite fast, as the mobility does not change when the sample is heated before loading. Variant ODN-2, forming an *i*M with 4 positive charges (S_1_), migrates slightly faster than *hras*-1^Y^, as expected. At pH 5, the 36-mer oligonucleotide containing sequence *hras*-2^Y^ migrates quicker than unstructured 27-mer ODN-1, as it folds into the *i*M (see CD). At pH 7, both *hras*-1^Y^ and *hras*-2^Y^ still migrate faster than ODN-1, indicating that even at neutral pH the sequences are folded. However, they migrate either with a smeared band (*hras*-1^Y^) or with two bands (*hras*-2^Y^), suggesting that more than one folded structure is formed: most likely *i*M-like structures stabilized by C:C and CH^+^:C bps. It is also possible that at pH 7, the *i*M-like structure is in equilibrium with a flexible hairpin (Fig. 2C, S_1_).

**Figure 3:**
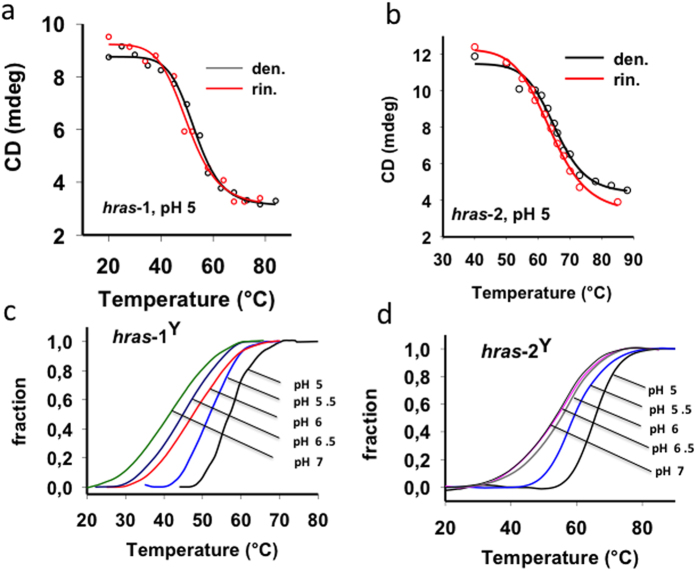
(**A,B**) CD melting curves of 3 μM *hras*-1^Y^ and *hras*-2^Y^ in 50 mM sodium cacodylate, pH 5, 50 mM KCl. Denaturing curve (20 → 85 °C), renaturing curve (85 → 20 °C); (**C,D**) Fraction of *i*M *versus* T curves obtained from FRET-melting experiment (0.3 °C/min), in 50 mM sodium cacodylate, pH 5 to 7, 50 mM KCl. The curves of *hras*-1^Y^ and *hras*-2^Y^ at pH 5 are fully reversible. FRET-melting gives *T*_M_ values ∼3 °C higher than CD-melting values, due to the presence of the fluorophores in the oligonucleotides analysed by FRET.

We have also examined the thermal stability of the *i*Ms by CD- and FRET-melting experiments. Typical CD-melting profiles for *hras*-1^Y^ and *hras*-2^Y^ are reported in Fig. 3A,B. By heating (20 → 85 °C) and then cooling (85 → 20 °C) the DNA solutions in 50 mM sodium cacodylate, pH 5, 50 mM KCl, the folded/unfolded transitions showed to be cooperative and reversible, as previously found for C-rich oligonucleotides under similar experimental conditions^10^,^12^,^13^,^14^. The melting in the pH range between 5 and 7 was examined by FRET experiments, using oligonucleotides end-labelled with ATTO-488 (5′-end) and TAMRA (3′-end) (Fig. 3C,D). The *T*_M_ of both *hras*-1^Y^ and *hras*-2^Y^ decreased with increasing pH: *hras*-1^Y^, 55.9, 52.1, 47.6, 44.8, 41.3 °C at pH 5, 5.5, 6, 6.5, 7, respectively; *hras*-2^Y^, 65.5, 58.7, 55.1, 53.8, 53.1 °C at pH 5, 5.5, 6, 6.5, 7. At pH values > 5, the *T*_M_ of the *i*Ms decreases as the structure is probably stabilized by a number of CH^+^:C < 6. It is indeed reasonable to assume that when the medium is not sufficiently acidic, the *i*M is stabilized by both C:C and CH^+^:C base pairs^36^,^37^. *i*M structures of different protonation levels may coexist in solution. Mechanical stability experiments with the ILPR C-rich sequence showed that partially folded *i*M-like species are in equilibrium with fully folded *i*M at neutral pH^36^. The higher *T*_M_ of *hras*-2^Y^ is likely due to the additional CH^+^:C bp that stabilizes the *i*M. The two structures have similar rupture forces and sufficient stability to stall RNA polymerase^36^. Yang and Rodgers have reported that the energy of C:C is about 1/3 of that of CH^+^:C^38^. Sequence *hras*-2^Y^ shows a behaviour similar to *hras*-1^Y^, with the difference that at pH 5 it shows a higher *T*_M_, 65.5 °C (Fig. 3D).

We evaluated the thermodynamics of the folding transitions according to a two-state model. This was done at pH 5, where the two sequences fold into only one structure, as shown by PAGE. From the CD- and FRET-melting curves at pH 5, we obtained the following average thermodynamic data (±10%): Δ*H* = 252 kJ/mol and Δ*S* = −770 J/mol K and Δ*G* = −17 kJ/mol for *hras*-1^Y^; Δ*H* = 323 kJ/mol and Δ*S* = −950 J/mol K and Δ*G* = −27 kJ/mol for *hras*-2^Y^. Assuming that the breaking of a CH^+^:C bp needs approximately 46 ± 4 kJ/mol^12^, the number of CH^+^:C broken by the thermal disruption of *hras*-1^**Y**^ is ~6, of *hras*-2^**Y**^ is ~7, in accord with the number of expected protons that should bind to the sequences at pH near pKa^10^,^12^. At pH 5, nearly half of the cytosines is protonated and the sequences are completely folded into *i*M, showing the highest stability.

### The *HRAS i*Ms are recognized by hnRNP A1

**Figure 4:**
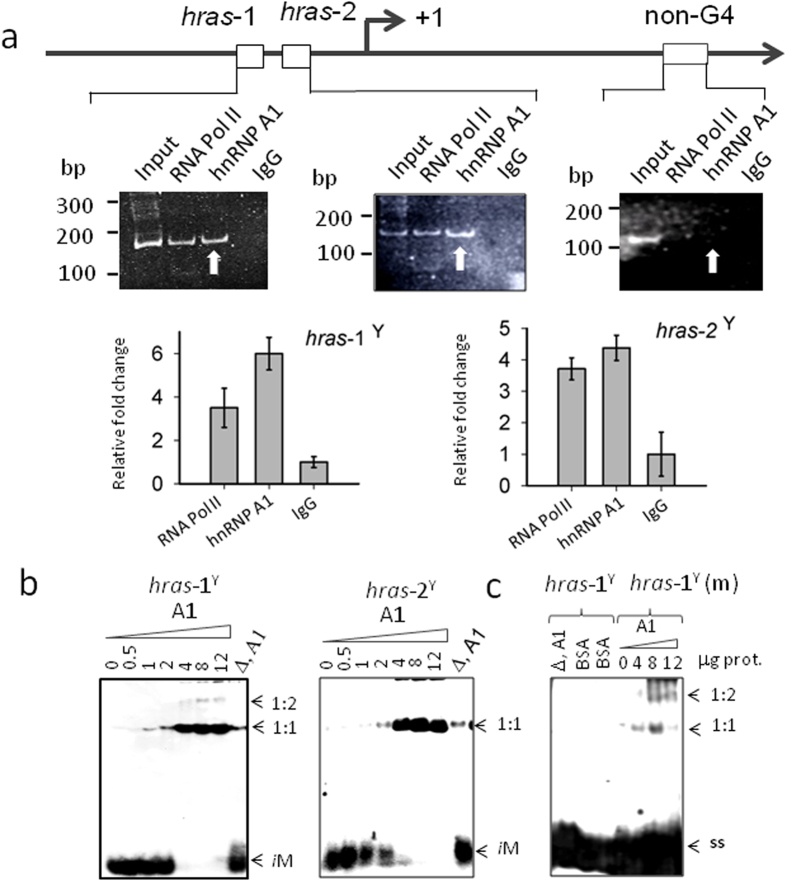
(**A**) ChIP experiment to determine the occupancy of *hras*-1, *hras*-2 and control sequence (870 bp downstream from TSS) by hnRNP A1. Histograms shows the relative occupancy of *hras*-1 and *hras*-2 by hnRNP A1, RNA Pol II (positive control) and IgG (negative control). Data have been normalized by IgG signal; (**B**) EMSA of ^32^P-labelled *hras*-1^Y^ and *hras*-2^Y^ in 50 mM Tris-acetate pH 5.5, 50 mM KCl, incubated 40 min at room temperature with increasing amounts of recombinant hnRNP A1 (0–12 μg). Lane (Δ,A1) indicates the *i*M incubated 40 min at room temperature, with denatured hnRNPA1 in binding buffer (see Methods); (**C**) EMSA at pH 5.5 of *hras*-1^Y^ with BSA or denatured hnRNP A1 and EMSA of *hras*-1^Y^ (m) with hnRNP A1; ss = single-stranded oligonucleotide; 1:1 and 1:2 DNA-protein complexes.

As the *i*M-forming sequences overlap critical GC-elements immediately upstream of TSS, we interrogated if these unusual structures are recognized by nuclear proteins. Previous studies have reported that DNA sequences composed by runs of cytosines such as the C-repeats in the telomeres and in the *CMYC* promoter are recognized by proteins of the heterogeneous nuclear riboproteins family (hnRNP)^39^,^40^ Moreover, Hurley and co-workers recently reported that the *i*M formed in the *BCL2* promoter interacts with hnRNP LL^6^. Specific binding of heterogeneous ribonuclear proteins to C-rich DNA sequences is also supported by previous work, according to which hnRNP A1 binds to the GC-element of *KRAS*^17^, which shows a high sequence/functional homology with the *HRAS* GC-elements. HnRNP A1 is one of the most abundant nuclear proteins of eukaryotic cells that regulates several aspects of mRNA biogenesis^41^. As it is over-expressed in a variety of cancers^41^,^42^, we wondered if this protein plays a role in the promoter of the *HRAS* oncogene, in the region where the *i*M can potentially be formed. To address this question, we first investigated by chromatin immunoprecipitation (ChIP) if in *HRAS*-mutant T24 bladder cancer cells, hnRNP A1 is associated to the GC-elements under *in vivo* conditions. The occupancy of *hras*-1 and *hras*-2 (located 6 bp upstream of first TSS) by hnRNP A1 was compared with the occupancy of a reference GC-rich sequence unable to fold into a non-B DNA structure (located 870 bp downstream from first TSS). A typical ChIP is shown in Fig. 4A. We found that the occupancy of *hras*-1 and *hras*-2 by hnRNP A1 was, respectively, ~6- and ~5-fold higher than the occupancy by IgG (negative control). As *hras*-1 and *hras*-2 are located in the region of the major transcription start sites, they show a significant occupancy by RNA Pol II: ~4-fold higher than the IgG signal. In contrast, the reference sequence showed almost no occupancy by any of the proteins considered. The ChIP data provided strong evidence that hnRNP A1, under *in vivo* conditions, is indeed associated to the critical GC-elements of the *HRAS* promoter. However, ChIP data do not provide information about the conformation of the GC-elements interacting with hnRNP A1. To know if the nuclear factor recognizes the *i*M, we performed EMSA at pH 5.5 and 20 °C of mixtures composed by *hras*-1^Y^ or *hras*-2^Y^ and recombinant hnRNP A1, which was produced with a high degree of purity (Fig. 4B,C). It can be seen that at pH 5.5 *hras*-1^Y^ and *hras*-2^Y^ (which are in the folded *i*M conformation) form with hnRNP A1 a retarded band due to a 1:1 DNA-protein complex. In the presence of 4 μg hnRNP A1, the *i*M is completely bound to the protein (Fig. 4B). With higher protein amounts, a second retarded band of much weaker intensity, probably a 1:2 complex, can be seen in the gel. When hnRNP A1 was thermally denatured before being added to the *i*M, the DNA-protein complex was abrogated and the *i*M migrated as a free molecule. As a further control, we used an unspecific protein like BSA and we found that it did not bind to the *i*M, as expected (Fig. 4C). When the *i*Ms was destabilized by replacing 4 C with 4 T (*hras*-1^Y^(m)) (see Methods), the binding was significantly attenuated, suggesting that the *i*M conformation is essential for optimal hnRNP A1 binding.

### hnRNP A1 unfolds the HRAS *i*-motif

**Figure 5:**
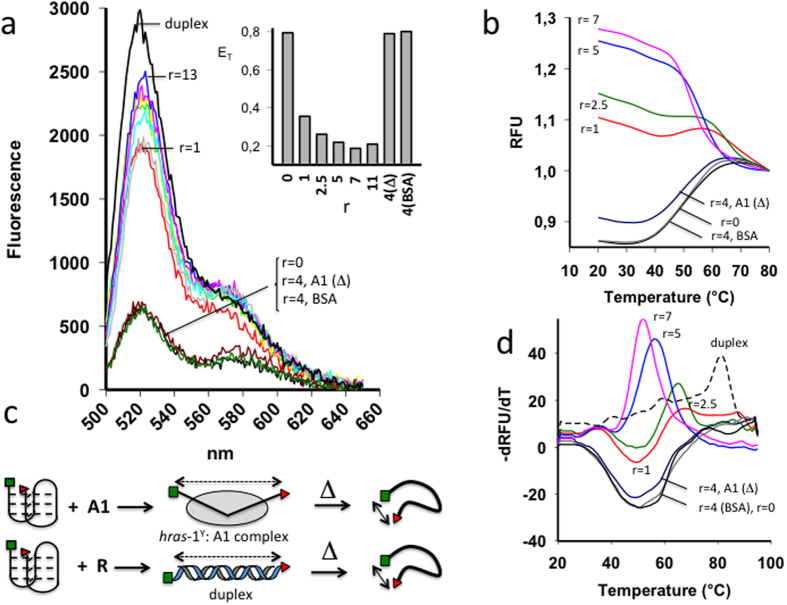
(**A**) FRET spectra of 200 nM hras-1^Y^ treated with increasing amounts of purified hnRNP A1 at pH 5.5, 50 mM sodium cacodylate, 50 mM KCl. As a control BSA and denatured hnRNP A1 (A1 Δ) have been used. Note that hnRNP A1 causes a dramatic increase of the 520 nm donor emission. The emission spectra of *hras*-1^Y^ hybridized to its complementary strand to yield the duplex is reported. Insight shows the energy transfer (*E*_T_) between donor-acceptor as a function of hnRNP A1 concentrations; (**B**) FRET-melting of *hras*-1^Y^ incubated with increasing amounts of hnRNP A1 (r = 1–7). The protein abrogates the melting profiles; (**C**) Cartoon showing the melting of *hras*-1^Y^ bound to its complementary strand or to hnRNP A1 (r = 1–7); (**D**) –dRFU/dT versus T curves of *hras*-1^Y^ alone, *hras*-1^Y^+protein, *hras*-1^Y^ in duplex, i.e. hybridized to its complementary strand.

In a previous work we have found that the *HRAS* promoter is highly active when the GC-elements are unfolded in the double-stranded conformation^3^,^4^. We now asked if the binding of hnRNP A1 to the *i*M involves the unfolding of this non B-DNA structure. To this purpose, we performed FRET experiments with *hras*-1^Y^ tagged with ATTO-488 (donor) and TAMRA (acceptor) in 50 mM sodium cacodylate, pH 5.5, 50 mM KCl. By exciting the donor at 480 nm, both donor (520 nm) and acceptor (580 nm) emit fluorescence, as a result of FRET between the fluorophores (Fig. 5A). When *hras*-1^Y^ is folded into the *i*M, the energy transfer, *E*_T_, between the two fluorophores is 0.77, and their end-to-end distance is ∼40 Å (S_2_). The effect of hnRNP A1 on the *i*M was investigated by incubating protein and DNA for 1.5 h and measuring the fluorescence between 500 and 650 nm, upon donor excitation at 480 nm. It can be seen that hnRNP A1 causes a dramatic increase of the donor emission, accompanied by a decrease of *E*_T_ as a function of r (r = [protein]/[*i*M]), in a dose-dependent manner, from 0.77 (r = 0) to 0.18 (r = 7) (inset). This means that the end-to-end distance in the *i*M increases from ~40 to ~64 Å, suggesting that upon binding to the protein, *hras*-1^Y^ goes through a conformational change. When *hras*-1^Y^ is in the duplex conformation, the fluorophores are separated by ~86 Å (26 × 0.33 = 86 Å, where 0.33 Å is the vertical rise per bp), as a 27-mer duplex behaves as an extended rigid rod. It follows that the *i*M bound to hnRNP A1 is not fully extended as in the duplex. As a control we used an unspecific protein as BSA and heated hnRNP A1 before incubation with the *i*M (20 min, 95 °C). In both cases the fluorescence of the donor did not increase, as expected (Fig. 5A).

To further support the finding that hnRNP A1 disrupts the *i*M, we carried out FRET-melting experiments, reasoning that the *i*M would not give its typical melting profile when bound to hnRNP A1. Fig. 5B shows that *hras*-1^Y^ *i*M in 50 mM sodium cacodylate, pH 5.5, 50 mM KCl has a *T*_M_ ~ 50 °C. In the presence of 4 equivalents (r = 4) of BSA or denatured hnRNP A1, the melting profile of *hras*-1^Y^ did not change, as expected. In contrast, when the *hras*-1^Y^ *i*M was incubated with increasing amounts of native hnRNP A1 (r = 1–7), its melting profile strongly changed, in keeping with the fact that *hras*-1^Y^ bound to the protein is not in the *i*M conformation. It is worth noting that free *hras*-1^Y^ melts with an increasing sigmoidal curve, whereas *hras*-1^Y^ bound to hnRNP A1 melts with a decreasing sigmoidal curve (Fig. 5B). A melting profile similar to that of the *hras*-1^Y^:hnRNP A1 complex is obtained with *hras*-1^Y^ in the duplex conformation with its complementary strand, where the two fluorophores are separated by ~86 Å. Upon melting, the duplex releases the *hras*-1^Y^ strand which, thanks to its flexibility, will have an end-to-end distance <86 Å (Fig. 5C). This results in a decreasing sigmoidal melting curve, and thus in a –dRFU/dT *versus* T curve marked by a positive peak. In the same way, *hras*-1^Y^ bound to hnRNP A1 is more extended than when it is free. Therefore, also the complex gives a decreasing melting curve and a first derivative curve with a positive peak at ∼50 °C. The melting of free *hras*-1^Y^ folded in the *i*M gives an increasing melting curve and a –dRFU/dT *versus* T curve with a negative peak (Fig. 5D). Summing up, both FRET titrations and melting provide strong evidence that hnRNP A1 unfolds the *HRAS i*M.

We have also analysed the effect of hnRNP A1 on the *hras*-2^Y^ *i*M. We found that the protein unfolds the *i*M of *hras*-2^Y^ at higher r values, as the *i*M formed by *hras*-2^Y^ has a higher stability than the *hras*-1^Y^ *i*M (58.7 *versus* 52.1 °C, at pH 5.5) (S_3_).

**Figure 6:**
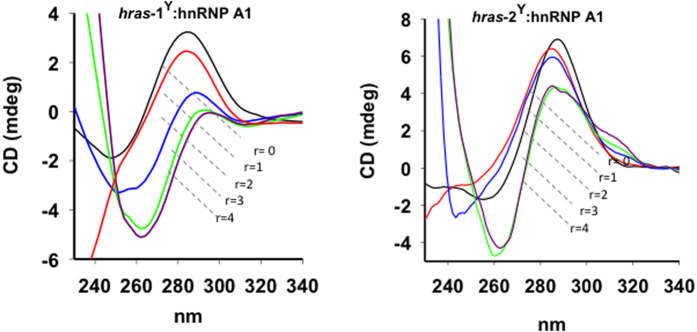
Circular dichroism analysis of 3 μM (0.5 cm pathlength cell) *hras*-1^Y^ and *hras*-2^Y^ at pH 5.5, 50 mM Tris-acetate, 50 mM KCl, after incubation with increasing amounts of hnRNP A1 (r = 0–4). Spectra of DNA-protein complex have been subtracted of protein spectrum.

The effect of hnRNP A1 on the *HRAS i*Ms was also investigated by CD (Fig. 6). At pH 5.5, *hras*-1^Y^ and *hras*-2^Y^ show the typical strong ellipticity at 287 nm of the *i*M conformation. When the *i*Ms are thermally denatured, the positive 287 nm ellipticity drops dramatically. This is a hallmark of the transformation of *i*M into ssDNA. A similar spectral change was obtained when we added increasing amounts of hnRNP A1 (*r* = 1, 2, 3, 4) to the *i*Ms. HnRNP A1 causes a progressive reduction of the 287 nm ellipticity, indicating that the *i*M structures are unfolded by the protein. As already observed with the FRET experiments, the unfolding effect is stronger with *hras*-1^Y^ than with *hras*-2^Y^, owing to the different stability of the two *i*Ms.

### Insights into the binding of hnRNP A1 to the *hras*-1^Y^ *i*M

**Figure 7:**
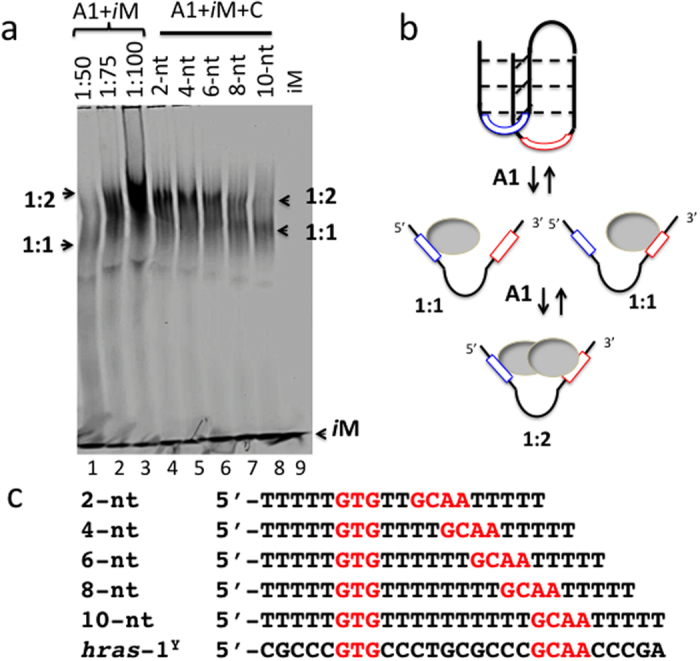
(**A**) 5% PAGE, lanes 4–8 show how the binding of hnRNP A1 to *hras*-1^Y^–dy781 (Methods) (ratio 1:100) at pH 5.5 is competed by oligonucleotides 2-nt - 10-nt (150-fold over *i*M). Lanes 1–3 shows the binding of *i*M to hnRNP A1 at ratios 1:50, 1:75, 1:100. Experimental conditions: DNA and protein incubated for 40 min at 37 °C, pH 5.5 before 5% PAGE analysis; (**B**) structure of the iM with the two lateral loops to which the protein is expected to bind; (**C**) sequences of the oligonucleotide competitors 2-nt, 4-nt, 6-nt, 8-nt, 10-nt.

Clues to the binding mode of hnRNP A1 to the *i*M can be obtained from the co-crystal structure of UP1 (the *N*-terminus of hnRNP A1 with DNA binding activity) and the human telomeric sequence d(TTAGGG)_2_^43^. The co-crystal shows that a protein dimer binds to two single-stranded strands, in the antiparallel orientation. As the two binding domains (RRM1 and RRM2) within each protein molecule are also antiparallel, the 5′ → 3′ polarity of ssDNA with respect to the RRM orientation is the same for each RRM. The two lateral loops of the *i*M may (after a minor adjustment) provide suitable binding sites for hnRNP A1, as they are antiparallel and separated by ~15–20 Å. So, the *i*M’s main function should be to provide a rigid chemical frame displaying two lateral loops with the precise nucleic acid directionality with respect to the RRM orientation. In other words, the *i*M structure should offer a kinetic advantage to the binding of hnRNP A1 (indeed, when the *i*M is disrupted, the binding is strongly attenuated, Fig. 4C). If we assume that the protein binds to the lateral loops, it can form either a 1:1 or a 1:2 complex, depending whether one or two protein molecules bind to the *i*M. The equilibria occurring in solution are: P + *i*M = P•iM (4); P + P•*i*M = (P)_2_•*i*M (5), where P is hnRNP A1. In the presence of a large excess of P compared to *i*M (1:100), both equilibria shift to the right forming complex 1:2. In contrast, with less P (ratio 1:50) equilibrium (5) does not shift to the right and only complex 1:1 is formed (Fig. 7A, lanes 1–3). In addition, as hnRNP A1 unfolds the *i*M, its binding depends on temperature: at 0 °C only complex 1:1 is formed probably because it requires a partial unfolding of *i*M, at 37 °C complex 1:2 is favoured as it requires a complete opening of the *i*M (S_4_). To support the binding of the protein to the lateral loops we performed the following competition experiment. As the two lateral loops of the *hras*-1^Y^ *i*M are separated by 10 nt, complex 1:2 should be competed by an oligonucleotide containing the two lateral-loop binding sites separated by a spacer of 10 nt (with a 10 nt spacer the competitor assumes a U-shape so that two antiparallel binding sites can interact with the protein RRMs). Moreover, if the spacer of the competitor is reduced to 8, 6, 4, and 2 nt, its capacity to compete with the formation of the 1:2 complex should gradually become weaker. To test this hypothesis we designed the competitors shown in Fig. 7C. When the competitors (150-fold in excess over *i*M) were incubated with the *i*M and hnRNP A1 (100-fold over *i*M), we found that the best competitor was the oligomer containing a spacer of 10 nt, which corresponds exactly to the distance of the lateral-loop binding sites in the wild-type *hras*-1^Y^ *i*M. These data support the binding of hnRNP A1 to the lateral loops of the *i*M, as observed for hnRNP LL and *BCL2 i*M^6^. As stated above, the *i*M facilitates the initial binding to hnRNP A1. Then, after the *i*M is unfolded, the protein should bind more stably to the *i*M sequence.

### HnRNP A1 knockout results in downregulation of *HRAS* transcription in human bladder cancer cells.

**Figure 8:**
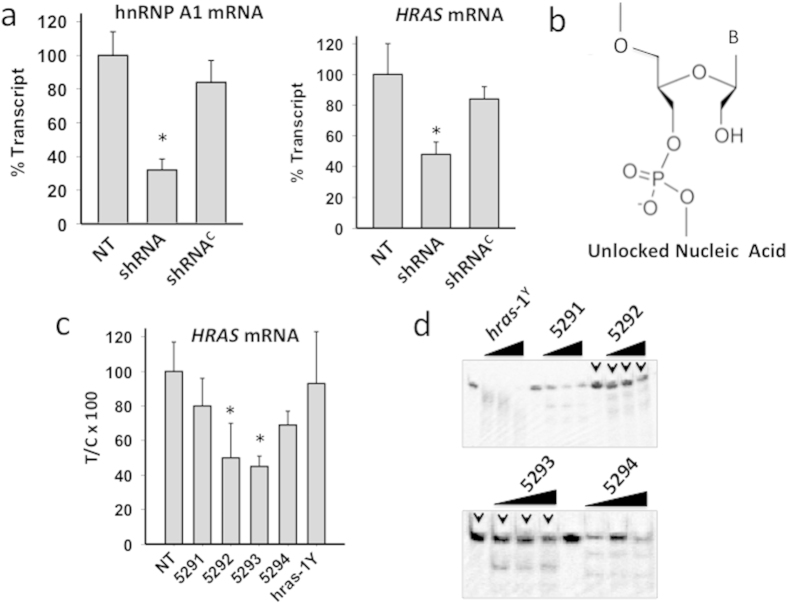
(**A**) Real-time determination of hnRNP A1 and *HRAS* mRNAs after knocking down hnRNP A1 in T24 bladder cancer cells with a specific shRNA. When hnRNP A1 is knocked down, *HRAS* mRNA is downregulated. P < 0.05 (*); (**B**) UNA modification introduced in the decoy oligonucleotides; (**C**) Level of *HRAS* mRNA in T24 cells treated with 200 nM *hras*-1^Y^ or UNA-modified analogues. Total RNA was extracted 24 h after oligonucleotide transfection, retro-transcribed and subjected to real time amplification. *HRAS* mRNA expression is normalized with housekeeping gene HPRT. The percentage of residual *HRAS* mRNA compared to *HPRT* mRNA in each sample is reported. P < 0.05 (*); (**D**) Resistance in fetal serum of *hras*-1^Y^ and UNA-modified analogues. Oligonucleotides have been incubated in serum for 0, 18, 24, 48 and 72 h at 37 °C. After incubation the samples have been run in denaturing PAGE, 7 M urea, 55 °C. The gels were stained with “stains all”.

As hnRNP A1 binds to the critical GC-elements of the *HRAS* promoter, we asked if the protein plays a role in transcription. We therefore evaluated the effect on transcription of knocking out hnRNP A1 by shRNA. First, we determined by quantitative real-time PCR the efficiency of shRNA to knock out hnRNP A1, finding that hnRNP A1 mRNA (normalized by the transcripts of β2-microglobulin and hypoxanthine guanine phosphoribosyltransferase, HPRT) was reduced to 29 ± 8% of the control (cells untreated or treated with a non-specific shRNA^C^), 48 h after treatment (Fig. 8A). In the same cells we also measured the level of *HRAS* mRNA finding that when hnRNP A1 is knocked out, the *HRAS* transcription is also down-regulated to 44 ± 5% of control. This suggests that hnRNP A1 is an essential factor for transcription, as previously reported^41^. Further evidence that the proteins recognizing the *i*Ms of *HRAS* are essential for transcription was obtained with decoy oligonucleotides mimicking *hras*-1^Y^ *i*M. These molecules, once introduced in the cells, should sequester the proteins (hnRNP A1 included) recognizing the C-rich strand of the *HRAS* GC-elements. To increase their nuclease resistance, the decoy oligonucleotides, namely **5291**–**5294** (see Methods), have been designed with unlocked nucleic acid (UNA) modifications (Fig. 8B) (S_5_)^44^,^45^. The capacity of the UNA-modified oligonucleotides to inhibit *HRAS* transcription was investigated by quantitative real-time PCR. T24 bladder cancer cells were transfected with the decoy oligonucleotides as well as with wild-type *hras*-1^Y^, using as transfecting agent jet-PEI^46^. After an incubation of 24 h, the total cellular RNA was extracted and the amount of *HRAS* mRNA relative to the housekeeping HPRT mRNA was evaluated by qRT-PCR (Fig. 8C). The results showed that oligonucleotides **5292** and **5293** reduced *HRAS* mRNA to ~50% of the control (untreated cells). We also examined by electrophoresis the nuclease resistance of the decoy oligonucleotides (Fig. 8D). The oligonucleotides were incubated in cell cultured medium containing 10% fetal bovine serum at 37 °C, pH 5.5 for 0, 18, 24 and 48 h. While *hras*-1^Y^ was quickly degraded, the UNA-modified oligonucleotides, in particular **5292** and **5293,** showed a remarkable stability, as the fraction of unbroken oligonucleotide was >0.5, after 48 h of incubation. Interestingly, the enhanced activity shown by these compounds correlates nicely with their higher stability in serum.

## Conclusion

We have demonstrated that two neighbouring GC-rich elements controlling *HRAS* expression can form non B-DNA *i*M structures, which are stable under near-physiological conditions. These unusual DNA structures are recognized by hnRNP A1, one of the most abundant nuclear proteins involved in the biogenesis of RNA. We have discovered that hnRNP A1 has a clear unfolding activity against the *i*M. As the knockout of hnRNP A1 by shRNA in T24 bladder cancer cells results in the inhibition of *HRAS*, hnRNP A1 behaves as an activating transcription factor. Our data, together with those of Hurley and co-workers, who showed that hnRNP LL binds to the *i*M of the *BCL2* promoter and activates transcription^6^, provide the first evidence that non B-DNA *i*M structures are recognized by nuclear proteins.

The proteins of the hnRNP family have been associated with the promoter of several genes where they are supposed to participate in the transcription regulation mechanisms, although their exact role is not yet fully understood. Some of them recognize C-rich sequences in the promoters of *CMYC* (hnRNP K)^39^,^40^, *BCL2* (hnRNP LL)^6^ and *HRAS* (hnRNP A1) (present study). These proteins seem to have a complex binding capacity, as hnRNP A1 is also able to bind to G-quadruplex DNA structures in *KRAS*^17^ and telomeres^47^. Remarkably, this type of binding is also associated with the disruption of G-quadruplex structures^17^.

Recent mechanical folding/unfolding experiments showed that G-quadruplex and *i*M are mutually exclusive within the same double-stranded tract^48^. However, whether this also holds under *in vivo* conditions, where double stranded DNA is exposed to negative superhelicity and located in a molecular crowding environment, has not yet been demonstrated. It is possible that both G-quadruplex and *i*M are extruded from each double-stranded GC-element, in the same way as two opposing hairpins (a cruciform) are extruded from a palindromic sequence. *HRAS* could therefore be regulated by a G-quadruplex/*i*M switch that represses transcription when the structural elements are in the folded conformation. Transcription will be activated when hnRNP A1 and MAZ, which recognize the *HRAS* G-quadruplexes^3^, bind to the *i*M and G-quadruplex, respectively, and then to other proteins of the transcriptional activator complex. These non B-DNA structures provide a mechanism for the control of gene expression at a different level than duplex, involving proteins recognizing these unusual structures that play a central role in gene regulation.

## Methods

### Oligonucleotides and hnRNP A1

The oligonucleotides used in this study have been obtained from Microsynth AG (Switzerland) and Eurofins Genomics (Germany):

5′-CGCCCGTGCCCTGCGCCCGCAACCCGA (*hras*-1^Y^)

5′-ACCGCGCGCCCCCGCCCCCGCCCCGCCCCGGCCTCG (*hras*-2^Y^)

5′-ATTO-CGCCGCCCGTGCCCTGCGCCCGCAACCCGAGC-TAMRA (A*-hras*-1^Y^-T)

5′-ATTO-CGC GCC CCC GCC CCC GCC CCG CCC C -TAMRA (A*-hras*-2^Y^-T)

5′-TCG GGT TGC GGG CGC AGG GCA CGG GCG (*hras*-1^R^)

5′-CGG GGC GGG GCG GGG GCG GGG GCG (*hras*-2^R^)

5′- CGC TCG TGC TCT GCG CTC GCA ACT CGA (*hras*-1^Y^m)

5′- TTTTTGTGTTTTTTTTTGCAATTTTT (ODN-1)

5′-CGTCCGTGTCCTGCGTCCGCAATCCGA (ODN-2)

5′-CGCCCGTGCCCTGCGCCCGCAGGGCGA (ODN-3)

5′-Dy 781-TTTTTTTCGCCCGTGCCCGTCGCCCGCAACCCGATTTTTTT-3′ (*hras*-1^Y^-dy 781). The oligonucleotides with UNA modifications have been synthesized in solid phase as previously described^44^,^45^: 5′-CGCCCGTGCCCTGuCGCCCGCuAACCCGuA (**5291**); 5′- CGCCCGTGCCCuUGCGCCCuGCAACCCGuA (**5292**); CGCCCGuUGCCCTGuCGCC-CGCuAACCCGuA (**5293**) and 5′-CGCCCGuUGCCCuUGCGCCCuGCAACCCGuA (**5294**) where uC, uU, uG, uA are unlocked nucleic acid nucleotides.

Recombinant hnRNP A1 tagged to GST was obtained with a high degree of purity as previously described^49^ (S_6_).

### Chromatin immunoprecipitation

T24 urinary bladder cancer cells (1.2 × 10^6^) were cultured overnight in 6-cm diameter plates up to about 80% confluency and fixed in 1% formaldehyde in PBS for 5 minutes at room temperature to crosslink proteins to DNA. Chromatin immunoprecipitation assays were performed using the ChIP-IT^TM^ Express kit (Active Motif, Rixensart, Belgium). Details are reported in S_7_.

### ShRNA transfection, RNA extraction and real-time PCR

T24 cells were plated in 96-well plate (10^4^ cells/well). After 1 day we transfected the cells with hnRNP A1-specific (sc-35576-SH) and control shRNA (sc-108066) (Santa Cruz, Dallas, USA) using as transfectant agent jetPEI^TM^ (Polyplus, NY, USA). After 48 h, RNA was extracted by using iScript ^TM^ RT-qPCR sample preparation reagent (BioRad, USA).

For cDNA synthesis, 1.25 μl of RNA was heated at 70 °C and placed in ice. The solution was added with 7.5 μl of a mix containing (final concentrations) 1 × buffer; 0.01 M DTT (Invitrogen); 1.6 μM primer dT [MWG Biotech, Ebersberg, Germany; d(T)_16_]; 1.6 μM random primers; 0.4 mM dNTPs solution containing equimolar amounts of dATP, dCTP, dGTP and dTTP (Euroclone, Pavia, Italy); 0.8 U/μl RNAse OUT; 8 U/μl of M-MLV reverse transcriptase (Life Technologies, Monza, Italy). The reactions were incubated for 1 h at 37 °C and stopped with heating at 95 °C for 5 min. As a negative control the reverse transcription reaction was performed with a sample containing DEPC water.

Real-time PCR multiplex reactions were performed with 1xKapa Probe fast qPCR kit for *HRAS* and housekeeping genes hypoxanthine-guanine phosphoribosyltransferase (HPRT) and β2-microglobulin, 2.2 μl of cDNA and primers/probes at the concentrations specified in S_1_. The PCR cycle was: 3 min at 95 °C, 50 cycles 10 s at 95 °C, 60 s at 58 °C. Real-time PCR amplification of hnRNP A1 was performed with 1 × Kapa Sybr Fast BioRad iCycle qPCR kit (KAPA Biosystems, Wilmington, MA, USA), 300 nM of each primer, 3.5 μl of cDNA (cycle: 3 min at 95 °C, 40 cycles 10 s at 95 °C, 30 s at 58 °C). PCR reactions were carried out with a CFX-96 real-time PCR apparatus controlled by an Optical System software (version 3.1) (Bio-Rad Laboratories, CA, USA). All expressions were normalized with housekeeping genes. The sequences of the primers and probes used for the amplifications are given as supplementary data (S_8_).

### CD and FRET experiments

CD spectra have been obtained with a JASCO J-600 spectropolarimeter equipped with a thermostatted cell holder. CD experiments were carried out with oligonucleotides *hras*-1^Y^ and *hras*-2^Y^ (3 μM) in 50 mM Tris-acetate, pH from 4.5 to 8, 50 mM KCl. Spectra were recorded in 1 or 0.5 cm quartz cuvette. A thermometer inserted in the cuvette holder allowed a precise measurement of the sample temperature. The spectra have been calculated with J-700 Standard Analysis software (Japan Spectroscopic Co, Ltd) and reported as ellipticity (mdeg) *versus* wavelength (nm). Each spectrum was recorded three times, smoothed and the baseline subtracted. CD spectra of 3 μM *hras*-1^Y^ and *hras*-2^Y^ have been obtained also at various temperatures (20-85 °C), by both heating and cooling the sample solutions (in 50 mM sodium cacodylate pH 5, 50 mM KCl). By plotting the 287 nm ellipticity versus temperature, sigmoidal denaturing and renaturing curves were obtained, which were practically overlapping.

FRET with oligonucleotides *hras-*1^Y^ and *hras*-2^Y^, tagged at the 5′ and 3′ ends with ATTO-488 and TAMRA (as donor we used ATTO-488 because its pH dependence is weaker than that of FAM), were carried out on a Microplate Spectrofluorometer System (Perkin Elmer 2300 Enspire, USA). Each sample contained 50 μl dual-labelled oligonucleotide (200 nM) in 50 mM Tris-acetate buffer, pH from 4.5 to 8, 50 mM KCl and an amount of hnRNP A1 as specified in the figure captions. The samples were incubated at 37 °C as specified in the text. Emission spectra were obtained setting the excitation wavelength at 480 nm and recording the emission from 500 to 650 nm. FRET-melting experiments of *hras-*1^Y^ and *hras*-2^Y^ have been performed on a real-time PCR apparatus (CFX96, BioRad, Hercules, CA) in 50 mM sodium cacodylate at pH 5, 5.5, 6, 6.5 and 7, 50 mM KCl. FRET-melting experiments were obtained by increasing the temperature from 20 °C to 95 °C (0.3 °C/min). From the melting data we obtained curves reporting the fraction of folded iM against temperature. These curves were reversible (denaturing and renaturing curves overlapping). The energy transfer (*E*_T_) was calculated from the fluorescence intensity of the donor D in the presence (*I*_DA_) and absence (*I*_D_) of the acceptor as:


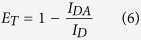


*I*_DA_ and *I*_D_ were measured in same buffer under identical concentrations (*I*_D_ was obtained by transforming the dual-labeled oligonucleotide into the corresponding duplex in which the fluorophores are at a distance for which FRET = 0). The FRET efficiency values were converted to distances between donor and acceptor by using:


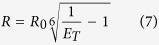


where *R* is the distance (Å) and *R*_0_ is the Föster distance [defined as the distance at which energy transfer is 50% of the maximum value, assumed to be 50 Å^50^].

### Thermodynamic analysis of reversible *i*M melting curves

The thermodynamic parameters for the folding of C-rich sequence into the *i*M conformation were obtained from the melting curves. From Δ*G*° = −RT ln *K* = Δ*H*°–*T*Δ*S*° (8) it is obtained ln*K* = (ΔH°/R)(1/*T*) + ΔS°/R (9). The equilibrium constant K as a function of T is given by *K* = *f*/(1–*f*), where *f*, the fraction of sequence folded in the *i*M conformation, is obtained form the melting curves. The plot of ln *K* versus 1/*T* gives a straight line whose slope (–Δ*H*/R) and y-intercept (–Δ*S*/R) provide the thermodynamic parameters (S_9_).

### PAGE assays

Oligonucleotides *hras*-1^Y^ and *hras*-2^Y^ were end-labelled with [γ-^32^P]ATP and T4 polynucleotide kinase. For competition experiments we used a DNA chemically labelled to dy-781. Before EMSA, the iM-forming oligonucleotides were allowed to form their structure in 50 mM Tris–acetate, pH 5.5, 50 mM KCl, (overnight incubation at room temperature). Radiolabelled oligonucleotides (10 nM) were incubated for 30 min at 20 °C with increasing amounts of hnRNP A1 (0–12 μg) as specified in Fig. 4B, in 50 mM Tris–acetate, pH 5.5, 50 mM KCl, 1 mM DTT, 8% glycerol, 1% Phosphatase Inhibitor Cocktail I (Sigma, Milan, Italy), 5 mM NaF, 1 mM Na_3_VO_4_, 2.5 ng/μl salmon sperm DNA (binding buffer). After incubation, the reaction mixtures were loaded in 5% PAGE in 50 mM Tris-acetate pH 5.5, thermostatted at 20 °C. After running the gel was dried and exposed to autoradiography (G E Healthcare, Milan) for 16 h at –80 °C. Mobility-shift experiments of cold *hras*-1^Y^ and *hras*-2^Y^ have been performed on 15% PAGE, 25 mM KCl, at pH 5 (50 mM sodium acetate) or pH 7 (50 mM Tris-acetate), 20 °C. 20% PAGE in denaturing 7 M urea conditions, was carried out in TBE. The gels were stained with “stains-all” dye. Competition assay with 28 nM *hras*-1^Y^-dy781 were performed at 37 °C with 3 μM of hnRNP A1 (100-fold over *i*M *hras*-1^Y^-dy781) and competitor oligonucleotides (150-fold over *i*M) in 50 mM Tris-acetate, pH 5.5, 50 mM KCl, 1 mM EDTA, 2.5ng/μl Salmon sperm. After incubation, the reaction mixtures were loaded in 5% PAGE 1xTBE, thermostated at 20 °C. After running the gel was analysed by Odyssey CLx scanner /ImageStudio Software (Li-Cor Biosciences).

### Cell culture and transfections

T24 human urinary bladder cancer cells were maintained in exponential growth in Dulbecco’s Modified Eagle’s Medium (DMEM) containing 100 U/ml penicillin, 100 mg/ml streptomycin, 20 mM L-glutamine and 10% fetal bovine serum (Euroclone, Milan, Italy).

For transfection we plated 10000 cells for each well in a 96 well plate and transfected using Jet PEI (Polyplus Illkirch FRANCE) following manufacturers *in vitro* protocol for DNA oligonucleotides transfection with 400 nM oligonucleotide (48 pmol) and N/P = 3.

## Additional Information

**How to cite this article**: Miglietta, G. *et al.* GC-elements controlling *HRAS* transcription form *i*-motif structures unfolded by heterogeneous ribonucleoprotein particle A1. *Sci. Rep.* **5**, 18097; doi: 10.1038/srep18097 (2015).

## Supplementary Material

Supplementary Information
