# Supplementary material for: GC-elements controlling HRAS transcription form i-motif structures unfolded by heterogeneous ribonucleoprotein particle A1
Source: Sci Rep. 2015 Dec 17;5:18097. doi: 10.1038/srep18097 (PMC4682182; doi:10.1038/srep18097)

**Supporting Information**

GC-rich elements controlling *HRAS* transcription form *i*-motif structures unfolded by heterogeneous ribonucleoprotein particle A1

Giulia Miglietta,*^a^*^,&^ Susanna Cogoi,*^a,&^* Erik B. Pedersen^b^ and

Luigi E. Xodo ^a*^

^a^ Department of Medical and Biological Sciences, P.le Kolbe 4, 33100 Udine, Italy;

^b^ Nucleic Acid Center, Institute of Physics and Chemistry, University of Southern Denmark, DK-5230 Odense M, Denmark.

**Supporting data S_1_**: Sequence of wild type *hras*-1^Y^ that form an *i*M in the pH range between 5 and 7. At pH ≥ 7 the sequence may form a hairpin stabilized by a stem with 5 W.C. base pairs. Sequence of mutant ODN-3 forming a hairpin stabilized by a stem of 7 base pairs. Sequence and possible structure of mutant ODN-2 forming an *i*M stabilized by 4 CH^+^:C base pairs.


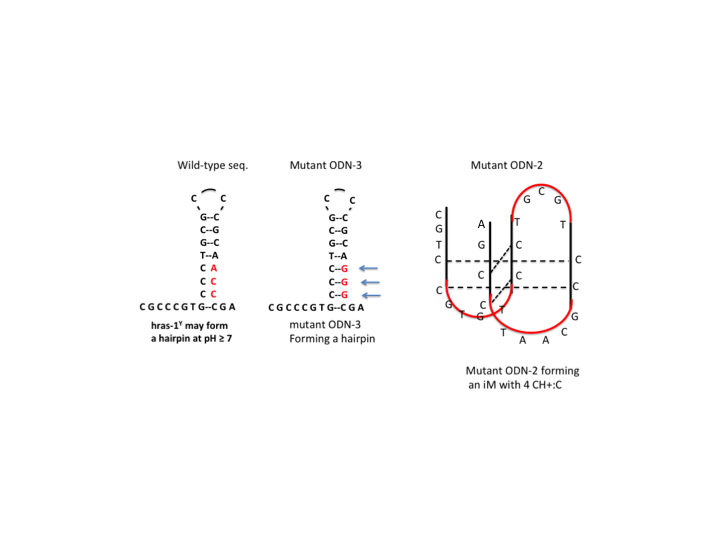


**Supporting data S_2_**: The inter-phosphate distance across the wide and narrow groove are respectively 1.5 and 0.7 nm (1-3). The high of the *i*M was estimated by assuming CH^+^:C stacking interval of 0.32 nm (4). The distance between two consecutive nt is assumed to be 0.43 nm (5). The end-to-end distance should be 7+4x4.3=24 Å, without taking into account the dyes. Considering that each dyes is conjugated at the 5’ and 3’ ends through a spacer, the end-to-end distance given by FRET, 40 Å, is consistent with the size of the *i*M.


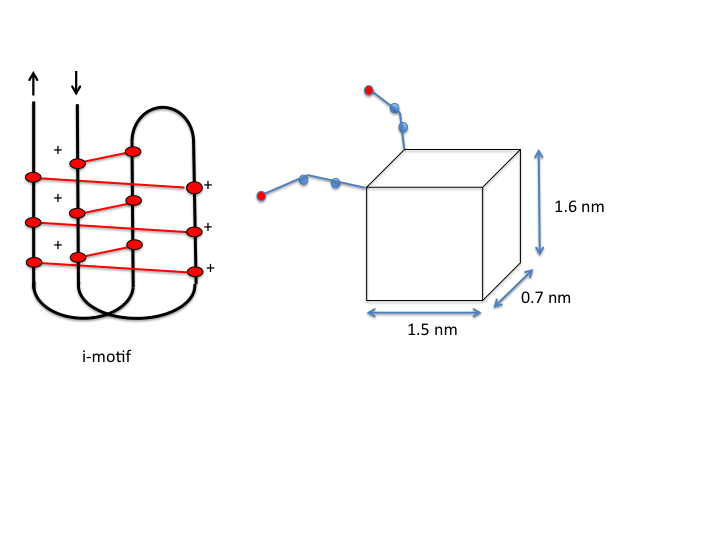


1. Mills, M.; Lacroix, L.; Arimondo, P. B.; Leroy, J. L.; Francois, J. C.; Klump, H.;  Mergny, J. L., *Curr. Med. Chem. - Anti-Cancer Agents* 2002, 2, 627-644.
2. Kang, C.; Berger, I.; Lockshin, C.; Ratliff, R.; Myozis, R.; Rich, A., *Proc. Nat. Acad. Sci.*  *USA* 1995, 92, 3874-3878.
3. Cai, L.; Chen, L.; Raghavan, S.; Ratliff, R.; Myozis, R.; Rich, A., *Nucleic Acids Res.*  1998, 26, 4696-4705.
4. Esmaili, N.; Leroy, J. L., *Nucleic Acids Res.* 2005, 33, 213-224.
5. [Dhakal](http://www.ncbi.nlm.nih.gov/pubmed/?term=Dhakal S%5Bauth%5D), S., [Schonhoft](http://www.ncbi.nlm.nih.gov/pubmed/?term=Schonhoft JD%5Bauth%5D), J.D., [Koirala](http://www.ncbi.nlm.nih.gov/pubmed/?term=Koirala D%5Bauth%5D), D., [Yu](http://www.ncbi.nlm.nih.gov/pubmed/?term=Yu Z%5Bauth%5D), Z., [Basu](http://www.ncbi.nlm.nih.gov/pubmed/?term=Basu S%5Bauth%5D), S. and [Mao](http://www.ncbi.nlm.nih.gov/pubmed/?term=Mao H%5Bauth%5D), H. (2010) [*J. Am. Chem. Soc*. 132, 8991–8997.](http://www.ncbi.nlm.nih.gov/entrez/eutils/elink.fcgi?dbfrom=pubmed&retmode=ref&cmd=prlinks&id=20545340)

**Supporting data S_3_:** (Left) FRET spectra of 200 nM *hras*-2^Y^ treated with increasing amounts of purified hnRNP A1 at pH 6.5, 50 mM sodium cacodylate, 50 mM KCl. As a control BSA and denatured hnRNP A1 (A1 Δ) have been used. HnRNP A1 causes a dramatic increase of the 520 nm donor emission. Insight shows the energy transfer (*E*_T_) between donor-acceptor as a function of hnRNP A1 concentrations; (B) FRET-melting of *hras*-2^Y^ incubated with increasing amounts of hnRNP A1. The protein abrogates the melting profiles.


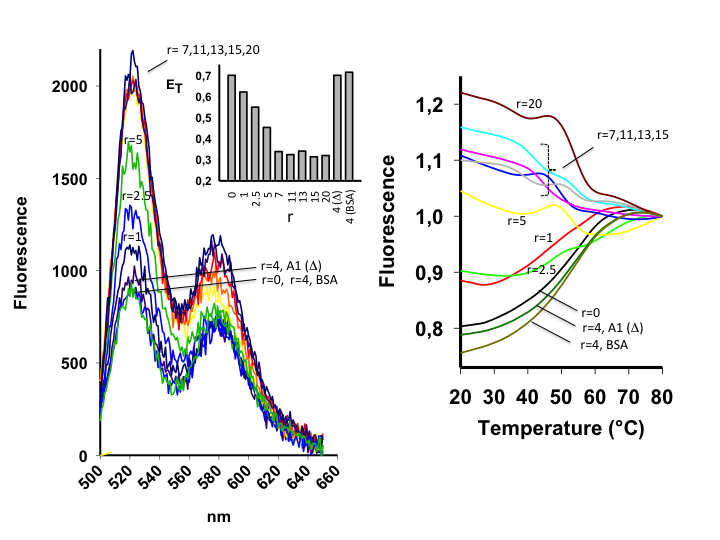


**Supporting data S_4_.** Binding of hnRNP A1 (100-fold) to the *i*M (28 nM) in binding buffer at pH 5.5 and T= 0, 20 and 37 °C. The iM sequence is 5’-Dy 781-TTT TTT TCG CCC GTG CCC GTC GCC CGC AAC CCG ATT TTT TT-3’ (*hras*-1^Y^-dy781). Before incubation with hnRNP A1 it was allowed to form its structure in 50 mM Tris-acetate pH 5.5 and 50 mM KCl. Then *hras*-1^Y^-dy781was incubated for 40 min at 0, 20 or 37 °C with 3 μM of hnRNP A1 (100-fold) in 50 mM Tris-acetate, pH 5.5, 50 mM KCl, 1 mM EDTA, 2.5ng/μl Salmon sperm. After incubation, the reaction mixtures were loaded in 5% PAGE 1 x TBE thermostated at 20°C. After running the gel was analysed by Odyssey CLx scanner and the shapes quantified by ImageStudio Software (Li-Cor Biosciences).


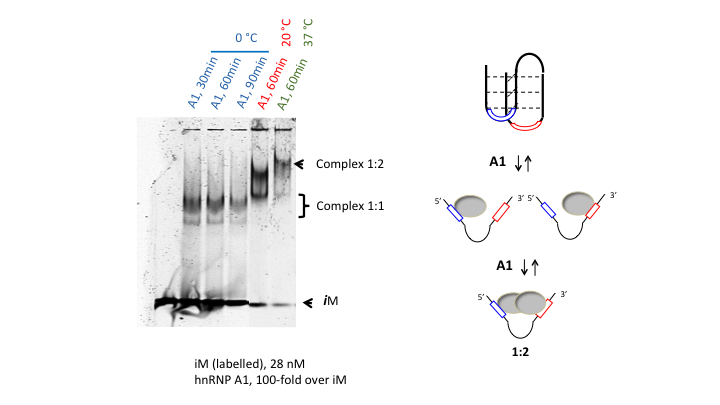


**Supporting data S_5_:** UNA decoy oligonucleotides**.**

The decoy oligonucleotides used are:

5’-CGCCCGTGCCCTGuCGCCCGCuAACCCGuA (**5291**);

5’- CGCCCGTGCCCuUGCGCCCuGCAACCCGuA (**5292**);

5’-CGCCCGuUGCCCTGuCGCCCGCuAACCCGuA (**5293**)

5’-CGCCCGuUGCCCuUGCGCCCuGCAACCCGuA (**5294**)

where uC, uU, uG, uA are unlocked nucleic acid nucleotides.

**A**

**
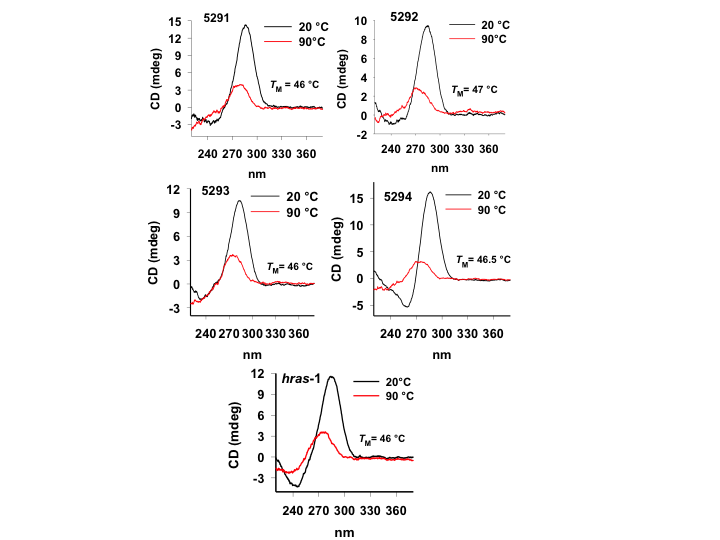
**

**B**

**
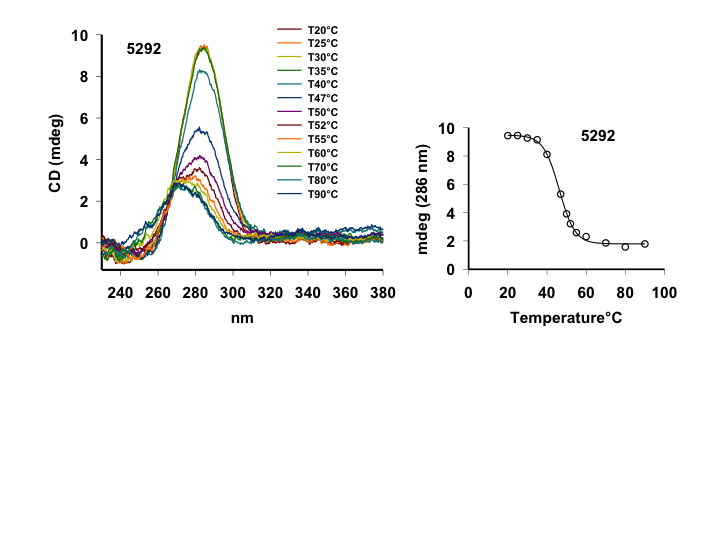
**

**(A)** Spectra of UNA-modified and wild-type *hras*-1^Y^ oligonucleotides (3 μM) at 20 and 90 °C in 50 mM cacodylate pH 5.5, 50 mM KCl. Note that the UNA modifications do not alter the structure of the *i*M. For each sequence we report the melting temperature obtained by CD spectra measured as a function of temperature. In panel B we show a typical CD-melting experiment obtained with oligonucleotide 5292.

**Supporting data S_6_: Recombinant hnRNP A1**

In brief, hnRNP A1 tagged to GST was expressed in *Escherichia coli* BL21 using pGEX-hnRNP

A1. After transformation, the bacteria were grown at 37°C in the presence of 50 μg/ml ampicillin up to an absorbance at 600 nm of 0.8 before the IPTG treatment (100 μM final concentration). The cells were allowed to grow for 5 h, harvested and centrifuged at 5000 r.p.m., 4°C. The supernatant was removed carefully and the pellet was re-suspended in a solution of PBS with 20 μM PMSF and 5 mM DTT. The bacteria were lysed by sonication (3 times 30 s, 20 kHz, Bioruptor, Diagenode, NY), added with Triton X-100 (1% final concentration) and incubated for 1.5 h on a shaker at room temperature. The lysate was then centrifuged for 10 min at 4°C at 10 000 r.p.m. Glutathione Sepharose 4B (GE Healthcare) (50% slurry in PBS) was added to the supernatant and incubated for 1.5 h at 4°C on a shaker. The mix was centrifuged for 5 min at 500 g and the pellet was washes 3 times in PBS and eluted with elution buffer containing 20 mM NaCl, 20 mM reduced glutathione,

200 mM Tris–HCl, pH 9.5. Finally, purification of hnRNP A1 was checked by SDS–PAGE.

**Supporting data S_7_: Chromatin Immunoprecipitaion**

T24 urinary bladder cancer cells (1.2 x 10^6^) were cultured overnight in 6-cm diameter plates up to about 80% confluency and fixed in 1% formaldehyde in PBS for 5 minutes at room temperature to crosslink proteins to DNA. Chromatin immunoprecipitation assays were performed using the ChIP-IT^TM^ Express kit (Active Motif, Rixensart, Belgium). The cells were lysed in 100 mM NaCl, 20 mM Tris-HCl, pH 7.4, 1 mM EDTA, 0.5 % NP-40, 0.5 % Na-deoxycholate, 0.1 SDS and sonicated to fragment chromosomal DNA into ~ 500 bp (8 min, 20 kHz, Bioruptor, Diagenode, NY). Sheared chromatin (6 μg in each sample) was diluted and incubated overnight, at 4 °C, with 0.5 μg of each specific antibody (Ab) and recovered with Protein G magnetic beads. The Abs used are hnRNP A1, (Sigma Aldrich), negative control mouse IgG (Active Motif), positive control RNA Pol II mouse monoclonal Ab (Active Motif). After incubation, the mixtures were spinned and the beads washed 3-times with kit washing buffers. The chromatin was treated with kit elution buffer for 15 min at RT on a shaker. We then used proteinase K (37 °C) to reverse the crosslinking and after 1 h we stopped the reaction with a proteinase K stop solution. The DNA recovered was amplified by PCR, using the following primers (accession number J00277): (i) 5’-GGCTCCTGACAGACGGG (304-320; *hras*-1for) and 5’-GCATGGGCTCCGTCC (477-491; *hras*-1rev) giving a 188 bp product; (ii) 5’-GGACGGAGCCCATGC (477-491; *hras*-2for) and 5’-CGTATTGCTGCCGCCT (624-639; *hras*-2rev) giving a 163 bp product. Amplification of control G-sequence (133 bp) downstream of the GC-elements was performed with: 5’-TCATTAAGAGCAAGTGGGGGC (1407-1428; control-for), 5’-CATCTGAAGGGCAAACCCACA (1518-1539, control-rev). Amplification products were separated by 8 % acrylamide gel in TBE and quantified with a Gel–DOC apparatus (Bio-Rad Laboratories, CA, USA). The data have been normalized by IgG.

**Supporting data S_8_: Sequences of primers and probes used in this study**

| **Accession Number** | **SENSE** | **ANTI-SENSE** | **PROBE** |
| --- | --- | --- | --- |
| NM_001130442  (HRAS) | **GCTGATCCAGAACCATTT**  (from 254 to 271) | **GTATCCAGGATGTCCAAC**  (from 344 to 361) | **HEX-CAATGACCACCTGCTTCCG-BHQ2**  (from 309 to 327) |
| NM_004048  (β2-microglobulin) | **CCCCACTGAAAAAGATGA**  (from 333 to 350) | **CCATGATGCTGCTTACAT**  (from 415 to 432) | **ROX-TATGCCTGCCGTGTGAACC-BHQ2**  (from 352 to 370) |
| NM_000194  (HPRT) | **CTTGATTGTGGAAGATATAATTG**  (from 557 to 575) | **TATATCCAACACTTCGTGG**  (from 672 to 690) | **Cy5-CTTGCGACCTTGACCATCTT-BHQ2**  (from 633 to 652) |
| NM_002136  (hnRNP A1) | **CATCGTTAAAGTCTCTCT TCAC**  (from 74 to 95) | **CAGGCTCTCATCAGTTGT**  (from 177 to 194) |  |

**Supporting data S_9_**: (Top panel) Denaturation profile of *hras*-2^Y^ in 50 mM sodium cacodylate pH 5, 50 mM KCl, obtained from FRET-melting experiments. The profile is reversible; (B) Typical thermodynamic analysis of the denaturation profile according to a two-state model. The parameters obtained are: ΔH= -338.2 kJ/mol, ΔS= -0.9 kJ/mol K, ΔG= -29.6 kJ/mol.


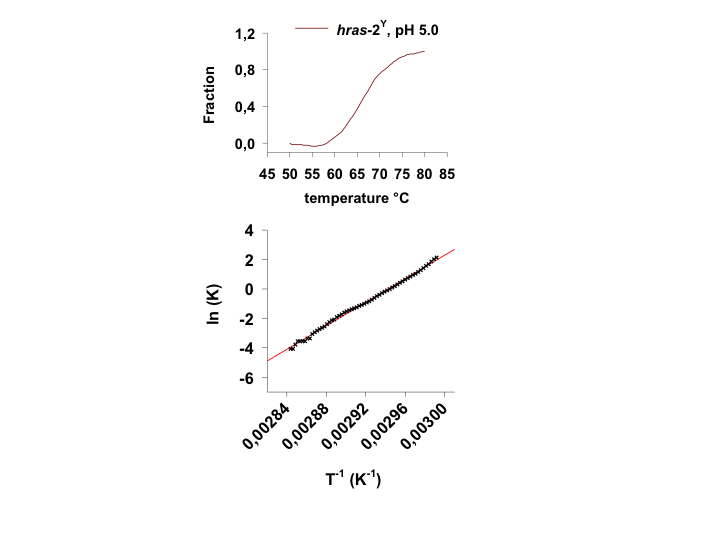

Supplement: Supplementary Information [file srep18097-s1.doc]
